# Tetrandrine reduces oxidative stress, apoptosis, and extracellular matrix degradation and improves intervertebral disc degeneration by inducing autophagy

**DOI:** 10.1080/21655979.2022.2031396

**Published:** 2022-02-02

**Authors:** Jintao Liu, Pengfei Yu, Feng Dai, Hong Jiang, Zhijia Ma

**Affiliations:** Department of Orthopaedic Surgery, Suzhou Tcm Hospital Affiliated to Nanjing University of Chinese Medicine, Suzhou, Jiangsu, PR China

**Keywords:** Tetrandrine, intervertebral disc degeneration, autophagy

## Abstract

Tetrandrine (TET) was reported to be an autophagy agonist, and the activating autophagy could delay intervertebral disc degeneration (IDD). Our study focused on exploring whether TET attenuated tert butyl hydrogen peroxide (TBHP)-induced nucleus pulposus (NP) cell injury and delayed rat IDD by inducing autophagy. In *vitro*, cytotoxicity was detected by MTT assay, ROS was measured with DCFH-DA probe, MDA, and SOD content was evaluated through ELISA, NP cell apoptosis was tested by flow cytometry, protein expression was detected by Western blot, in particular, LC3 expression was assessed by immunofluorescence. In *vivo*, pathological changes were estimated by HE and safranin-O staining, related protein expression was measured by immunohistochemistry, and the apoptosis was detected by TUNEL. Compared with the control group, oxidative stress, apoptosis, and extracellular matrix (ECM) degradation were increased, the expression of cleaved caspase-3,9, aggrecan and collagen II were reduced, and the expression of MMP13 and ADAMTS5 were up-regulated in TBHP-treated NP cells. Moreover, TET could reverse the effect of TBHP on NP cells. Further, TET enhanced autophagy in NP cells by amplifying the LC3 II/LC3 I/ratio and reducing p62 expression, which attenuated oxidative stress, apoptosis, and ECM degradation in TBHP-treated NP cells. In addition, in *vivo*, TET delayed rat IDD, increased the expression of LC3 and collagen II, and weakened apoptosis. TET inhibited oxidative stress, apoptosis, and ECM degradation in TBHP-treated NP cells by inducing autophagy, and alleviated IDD. These indicated that TET might be a potential candidate drug for the treatment of IDD.

## Graphical abstracts

**Figure:**
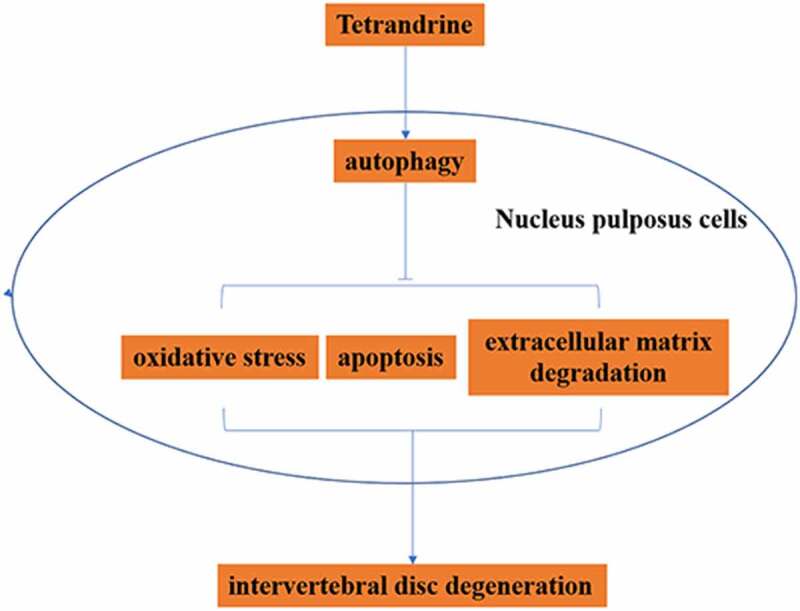

## Introduction

Intervertebral disc (IVD) is a special structure located between two adjacent cones of human spine, which is composed of annulus fibrosus (AF), nucleus pulposus (NP) and upper and lower cartilage endplates [1,2]. Main function of IVD is to alleviate the stress caused by cone compression, rotation, and tension, so as to ensure well mobility of human spine [2,3]. However, with the increase of age, intervertebral disc degeneration (IDD) will occur a series of degenerative diseases [4]. Low back pain (LBP) is the most general disease caused by IDD [4], and which bring serious burden to the family and society. At present, the exact pathogenesis of IDD is still not clear, so its treatment was only limited to alleviating symptoms through surgery. Therefore, it is very important to study the molecular mechanism of IDD.

Most researchers believe that the common cause of IDD is the imbalance of cell metabolism caused by biomechanical abnormalities and nutritional dysfunction [5]. In recent years, researchers gradually found that IVD cells regulate the homeostasis of IVD environment by secreting extracellular matrix (ECM) and related regulatory factors (matrix metalloproteinases (MMPs) and a disintegrin and metalloprotease with thrombospondin motifs (ADAMTSs)) [6,7]. Therefore, the function of IVD is directly affected by synthesis of ECM.

Both apoptotic and autophagic cell death belong to programmed cell death, and they affect each other under some stimuli [8,9]. It has been reported that the balance of autophagy and apoptosis of NP cells is closely related to IDD [10–12]. Jiang et al. pointed out that the number of autophagosomes in NP cells of patients with IDD was significantly decreased in compare with normal people [13]. Miyazaki et al. showed that SIRT1, as an effective therapeutic target of IDD, could protect against mitochondrial apoptosis induced by nutritional deprivation through autophagy induction in human NP cells [14]. The study found that autophagy could inhibit apoptosis in IDD, and spermidine produced a beneficial effect on IDD through autophagy stimulation [15]. In NP cell induced by tert butyl hydrogen peroxide (TBHP), autophagy activation of NP cells could reduce apoptosis, increase the expression of collagen II, and reduce the expression of MMP13 and ADAMTS-4, 5. However, 3-MA blocking autophagy promoted the apoptosis of NP cells [16]. Therefore, autophagy activation protects NP cells from apoptosis and improves disc homeostasis, which may be beneficial to the treatment of IDD. Tetrandrine (TET) is a dibenzyl isoquinoline alkaloid, which has been proved to be a calcium channel blocker and calmodulin antagonist [17]. TET is involved in anti-inflammatory, anti-cancer, reversal of multidrug resistance, induction of autophagy and so on [17,18]. TET attenuates the bone erosion in collagen-induced arthritis rats by inhibiting the translocation of NF-κB-p65 and NFATc1 [19]. TET alleviated complete Freund’s adjuvant-induced foot swelling, synovial inflammation, and pro-inflammatory cytokines secretion, and showed ain-inflammatory effect in LPS-induced macrophage RAW 264.7 cells and chondrogenic ATDC5 cells through inhibiting IκBα and NF-κB p65 phosphorylation [20]. However, whether TET could improve IDD has not been reported.

In this study, we aimed to explored the effect of TET on IDD. It was hypothesized that TET could improve IDD through activating autophagy. we used TBHP, a stable form of hydrogen peroxide, to trigger oxidative stress, which is widely accepted as an in vitro model to induce ECM degeneration and the apoptosis of NP cells [21,22]. In addition, we used TBHP to induce an oxidative microenvironment because it offers several advantages over hydrogen peroxide (H2O2), such as high stability and slow release [23].

Moreover, the animal model was constructed through AF puncture. Then, the effects of TET on IDD in *vivo* and in *vitro* were explored. Our study showed that TET decreased oxidative stress, apoptosis, and ECM degeneration through inducing autophagy in IDD. These data suggested that TET may be a potential candidate for the treatment of IDD.

## Materials and methods

### Cell culture and treatment

Human NP primary cells (No.: CSI113Hu01) were purchased from CLOUD-CLONE CORP. WUHAN. The cells came from the nucleus pulposus of normal people, and did not contain HIV-1, HBV, HCV, mycoplasma, bacteria, yeast, fungi, etc. The cells were cultured in DMEM/F12 (1:1) mixed medium (Gibco, USA) containing 10% fetal bovine serum (Invitrogen, USA) and 1% streptomycin (Gibco, USA). The culture conditions were 37°C and 5% CO_2_. The third passage of NP cells were used in subsequent experiments.

In order to study the toxicity of TET and TBHP (Sigma-Aldrich, USA) on NP cells, cells were treated for 12 h, 24 h, 48 h with TET (0, 0.01, 0.05, 0.1, 0.5, 1, 5, 10 μM), and TBHP (0, 10, 25, 50, 75, 100, 125, 150 μM) were used to treat cells for 24 h. As previous study [22], in order to study the effect of TET on TBHP-treated NP cells, the cells were pretreated for 2 h with TET (0, 0.1, 0.5, 1 μM); then, treated with TBHP (75 μM) for 24 h, and the same amount of normal saline was used as the control group. To explore the role of autophagy in the protective effect of TET on IDD, NP cells were pretreated for 2 h with 3-MA (5 mM) or rapamycin (100 nM) (Selleck Chemical, USA), and then treated with TET and/or TBHP.

### MTT assay

NP cells in logarithmic growth stage were inoculated into 96-well plates according to 1 × 10^4^ cells per well. After 24 h of culture, the cells were treated according to the above description. After another 24 h of culture, 20 μl of 5 mg/ml MTT solution (Beyotime, China) was added to each well. After 4 h of incubation, the supernatant was discarded, and DMSO (150 μl) (Beyotime, China) was added to each well and mixed for 2 min. The absorbance value at 570 nm wavelength of each well was detected by a microplate reader (BioTek, USA).

### Flow cytometry

The NP cells were intervened as previously described. The NP cells were collected, washed with precooled PBS for three times, centrifuged at 1000 rpm for 5 min, and prepared into cell suspension (1 × 10^6^ cells/ml) using binding buffer. 5 μl Annexin V/FITC (BD Biosciences, USA) and 10 μl PI (BD Biosciences, USA) were mixed with the cells, and then the cells were incubated in the dark at room temperature for 15 min. The apoptosis was measured with FACSCalibur flow cytometer (BD Biosciences, USA).

### Western blot

The cells were lysed for 30 min using RIPA reagent (Beyotime, China) containing protease inhibitors (Beyotime) on ice, and then the supernatant was collected by centrifugation (12,000 rpm, 5 min, 4°C). The protein concentration in the supernatant was detected by BCA detection kit (Beyotime, China). 25 μg protein per well was subjected to SDS-PAGE and then electrically transferred to PVDF membranes (Millipore, USA). The membranes were sealed with 5% skimmed milk powder for 1 h, the corresponding primary antibody [cleaved caspase 3 (1:1000, ab2302, Abcam), cleaved caspase 9 (1:1000, ab2324, Abcam), aggrecan (1:1000, ab3778, Abcam), collagen II (1:1000, ab34712, Abcam), MMP13 (1:1000, ab51072, Abcam), ADAMTS4 (1:1000, ab185722, Abcam), LC3A/LC3B (1:1000, PA1-16,930, Invitrogen), p62 (1:1000, PA5-20,839, Invitrogen), GAPDH (1:2000, ab8245, Abcam)] was added and incubated overnight at 4°C, and the secondary antibody [goat anti-rabbit (1:2000, ab150077, Abcam), goat anti-mouse (1:2000, ab150113, Abcam)] was added and incubated at room temperature for 2 h, the protein bands were developed with ECL reagent (Beyotime), and the gray value was quantified using image J 6.0 (NIH).

### ELISA assay

NP cells were seeded in a 96-well plate at a density of 1 × 10^4^ cells/well. After being pre-treated with TET for 2 h, the cells were exposed to 24 h of TBHP. Next, the NP cells were collected through centrifugation (12,000 × g, 4°C, 10 min). Cells were treated with RIPA lysis buffer, and then the supernatant was extracted through centrifugation (12,000 × g, 4°C, 30 min). The content of ROS (E004-1-1), MDA (A003-1-2), and SOD (A001-3-2) in NP cells was assayed according to the instructions of ELISA kit (Nanjing Jiancheng Bioengineering Institute, China).

### Immunofluorescence

The NP cells of each group were inoculated into 24 well plates at a density of 2.5 × 10^4^/well and cultured for 24 h. Then, the cells were fixed for 15 min with 4% paraformaldehyde (Beyotime), treated for 20 min with 0.5% Triton X-100 (Beyotime), blocked for 30 min with goat serum (Beyotime) at room temperature, and then incubated overnight with antibodies to LC3 (1:200) at 4°C overnight. After the fluorescent secondary antibody (1:200) was added and incubated at room temperature for 2 h, 4′,6-diamidino-2-phenylindole (DAPI, Beyotime) was added for 5 min, and then a fluorescence microscope (Olympus Corporation, Japan) was used to observe the expression of LC3. The results were analyzed with Image J 6.0 (NIH).

### IDD rat model

Eighteen male Sprague-Dawley rats (8-week-old) were obtained from Shanghai Lab. Animal Reasearch Center, which were randomly divided into control group (n = 6), IDD model group (n = 6) and TET group (n = 6). After adaptive feeding for 1 week, the rats were anesthetized and used 3% pentobarbital sodium (30 mg/kg) to puncture the AF of IVD from the external posterior abdominal approach to establish the IDD model. Taking 2–3 cm long incision in the right ventral posterolateral, a 21 G puncture needle was used to conduct full-thickness acupuncture on the AF of L5/6 IVD, and the acupuncture depth was 2.3 mm. The needle was pulled out after maintaining for 10s. Then, the peritoneum, muscle and skin were sutured. Rats were injected with penicillin 80,000 U/day for 3 consecutive days. The next day after operation, the rats were administered with 40 mg/kg TET per day by gavage. After two weeks of treatment, the rats were euthanized by intraperitoneal injection of excessive anesthetic. The animal study was performed according to the Guide for the Care and Use of Laboratory Animals of Suzhou Hospital of Traditional Chinese Medicine, and approved by the Ethical Commission of Suzhou Hospital of Traditional Chinese Medicine.

### HE staining

The IVD tissue was fixed with 4% paraformaldehyde (Solarbio, China), embedded in paraffin and made into 5 μm sections. After dewaxing and gradient ethanol hydration, the sections were stained with hematoxylin (Solarbio, China) for 5 min, differentiated with hydrochloric acid ethanol for 30 s, immersed with warm water for 15 min, stained with eosin (Solarbio, China) for 2 min, dehydrated with gradient ethanol, and then sealed with neutral resin. The damage of NP and AF were observed under the microscope.

### Safranin-O staining

The sections were dewaxed, hydrated with gradient ethanol, stained with 0.5% safranin-O (Solarbio, China) for 30 min, differentiated with 95% hydrochloric acid alcohol for 30 s at room temperature, dehydrated with gradient alcohol, vitrificated by dimethylbenzene and sealed with neutral resin. The results were analyzed by image J 6.0 (NIH).

### Immunohistochemistry

After dewaxing and hydration, IVD tissue sections were soaked in xylene for 30 min. The sections were boiled in sodium citrate buffer for 30 min for antigen repair. The sections were blocked for 30 min with goat serum (Beyotime), then reacted with the primary antibody overnight at 4°C, and then incubated with the secondary antibody for 30 min at room temperature. Following that, DAB reaction was used for 10 min in dark. After cleaning, hematoxylin was used to re-stain the nucleus. After dehydration, vitrification and sealing, the results were observed and photographed under the microscope. The positive expression of LC3 and collagen II was yellow brown. Image J was used to analyze the results.

### TUNEL assay

After dewaxing and hydration, the IVD tissue sections were reacted with TUNEL reaction mixture at 37°C for 1 h in dark, then stained with DAPI for 5 min, and the apoptosis was observed under a fluorescence microscope (Olympus, Fluoview1000, Tokyo, Japan).

### Statistical analysis

SPSS 18.0 software (SPSS Inc., USA) was used for statistical analysis. The measurement data were expressed by mean ± standard deviation (SD), one-way ANOVA followed by Tukey’s multiple-comparison *post hoc* test was used for comparison among groups, and the difference was statistically significant (*P*< 0.05). At least three independent experiments were performed.

## Results

### The effect of TET on the activity of NP cells

**Figure 1.**
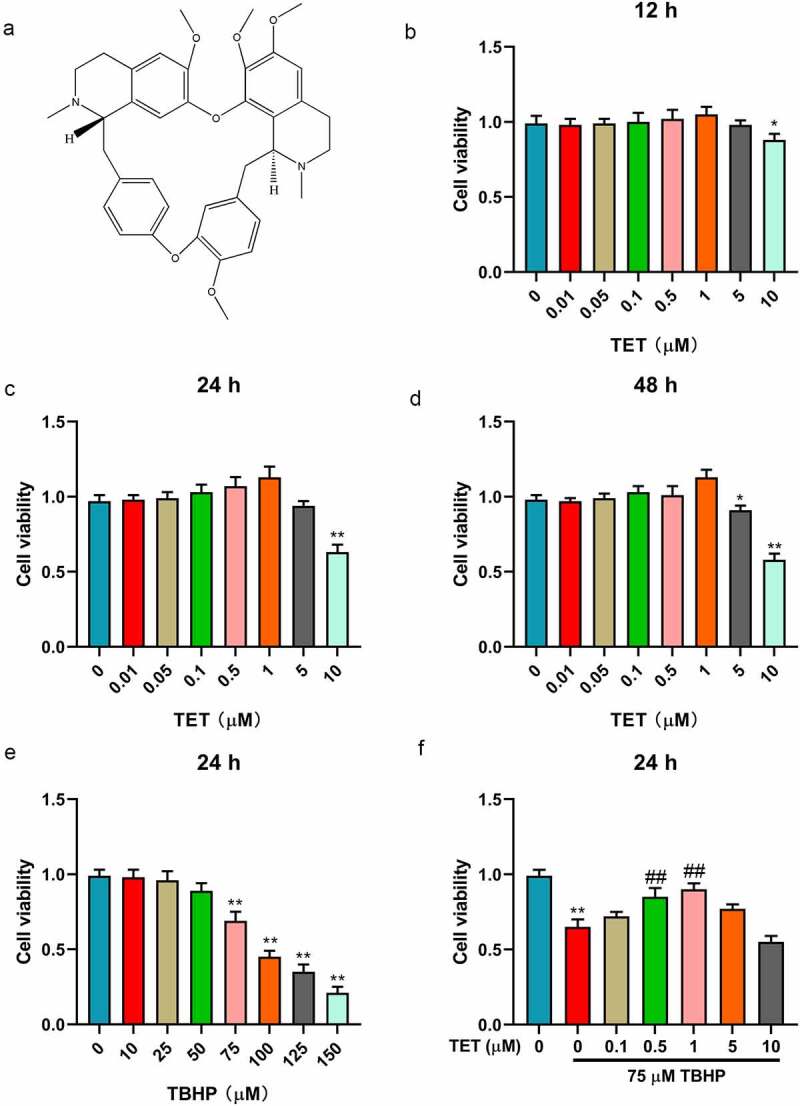
The effect of TET and TBHP on NP cells viability. (a) The chemical structure of TET was shown. (b-d) After treatment with TET (0, 0.01, 0.05, 0.1, 0.5, 1, 5, 10 μM) for 12 h, 24 h, 48 h, NP cells viability was measured by MTT assay. (e) After treatment with TET (0, 10, 25, 50, 75, 100, 125, 150 μM) for 24 h, NP cells viability was detected by MTT assay. (f) After treatment with TET (0, 0.1, 0.5, 1, 5, 10 μM) for 2 h, NP cells were administered for 24 h with TBHP (75 μM), the same amount of normal saline was used as control, and then the cell viability was tested by MTT assay. *p < 0.05 vs. control group, **p < 0.01 vs. control group, ##*p* < 0.01 vs. TBHP group.

To explore the cytotoxic of TET on NP cells. The structural formula of TET was analyzed through PubChem database (https://pubchem.ncbi.nlm.nih.gov/compound/73078) (Figure 1a). The cytotoxic of TET or/and TBHP was detected through MTT assay. The results showed that TET had no toxicity to NP cells in the concentration range of 0–1 μM after 12 h, 24 h, and 48 h treatment (Figure 1B,D). TBHP (≥50 μm) could significantly inhibit the activity of NP cells, and 75 μm TBHP was selected for follow-up experiments (Figure 1e). Importantly, pre-treatment of TET (0.1–1 μm) could attenuate the toxicity of TBHP to NP cells (figure 1f).

### TET inhibited TBHP induced oxidative stress and apoptosis of NP cells

**Figure 2.**
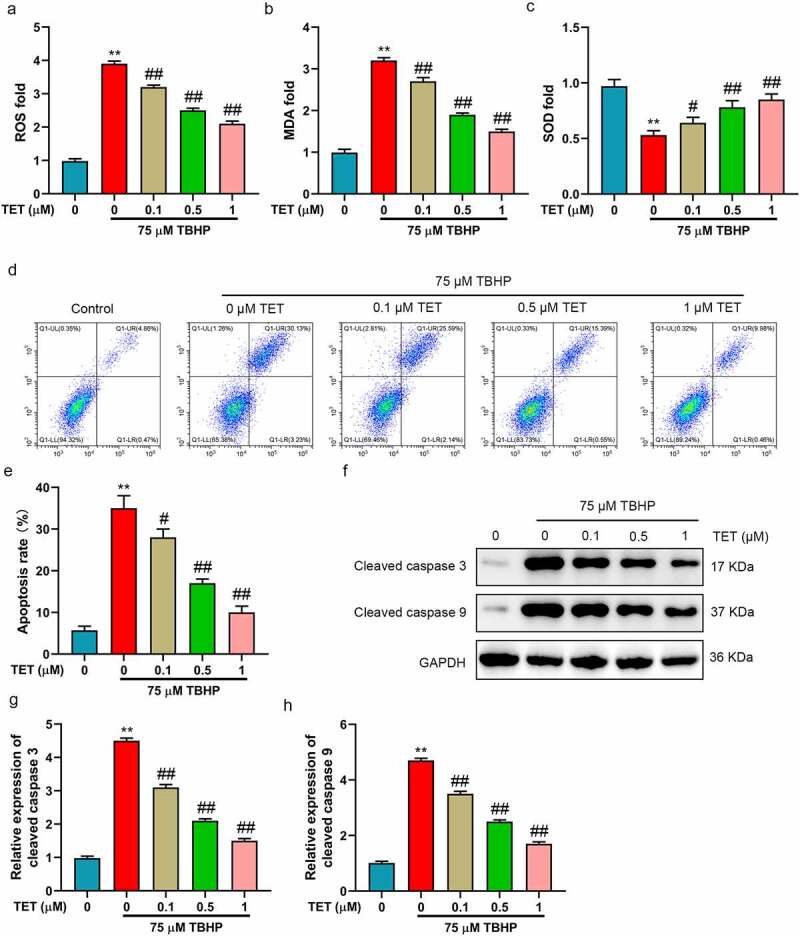
TET reduced oxidative stress and apoptosis in TBHP-treated NP cells. After treatment with TET (0, 0.1, 0.5, 1, 5, 10 μM) for 2 h, NP cells were administered for 24 h with TBHP (75 μM), and the same amount of normal saline was used as control. (a-c) ROS, MDA and SOD contents were evaluated by ELISA. (d) NP cells apoptosis was measured by flow cytometry. (e) Apoptosis rate was showed in a histogram (f) Cleaved caspase 3 and cleaved caspase 9 expression were tested by Western blot. (g-h) THE relative expression of cleaved caspase 3 and cleaved caspase 9 were showed in histograms. **p < 0.01 vs. control group, #*p* < 0.05 vs. TBHP group, ##*p* < 0.01 vs. TBHP group.

Oxidative stress and apoptosis are important markers of IDD [24]. The effect of TET on oxidative stress and apoptosis was researched in TBHP-treated NP cells. In this study, the level of ROS was increased in TBHP-treated NP cells, and which was partially inhibited by TET pre-treatment with a concentration dependent (Figure 2a). The content of MDA was higher in TBHP-treated NP cells than in the control group (Figure 2b), while SOD was decreased (Figure 2c). TET reversed the effects of TBHP on MDA and SOD in NP cells (Figure 2b,c). Subsequently, the results of flow cytometry indicated that TBHP treatment resulted in increased apoptosis of NP cells. Interestingly, and TBHP-induced apoptosis was gradually weakened with the increase of TET concentration (Figure 2d,e). In addition, TET prevented the increase of cleaved caspase-3 and cleaved caspase-9 (apoptosis marker proteins) induced by TBHP (Figure 2f,h). These results illustrated that TET attenuated oxidative stress and apoptosis in NP cells induced by TBHP.

### TET reversed TBHP induced ECM reduction in NP cells

**Figure 3.**
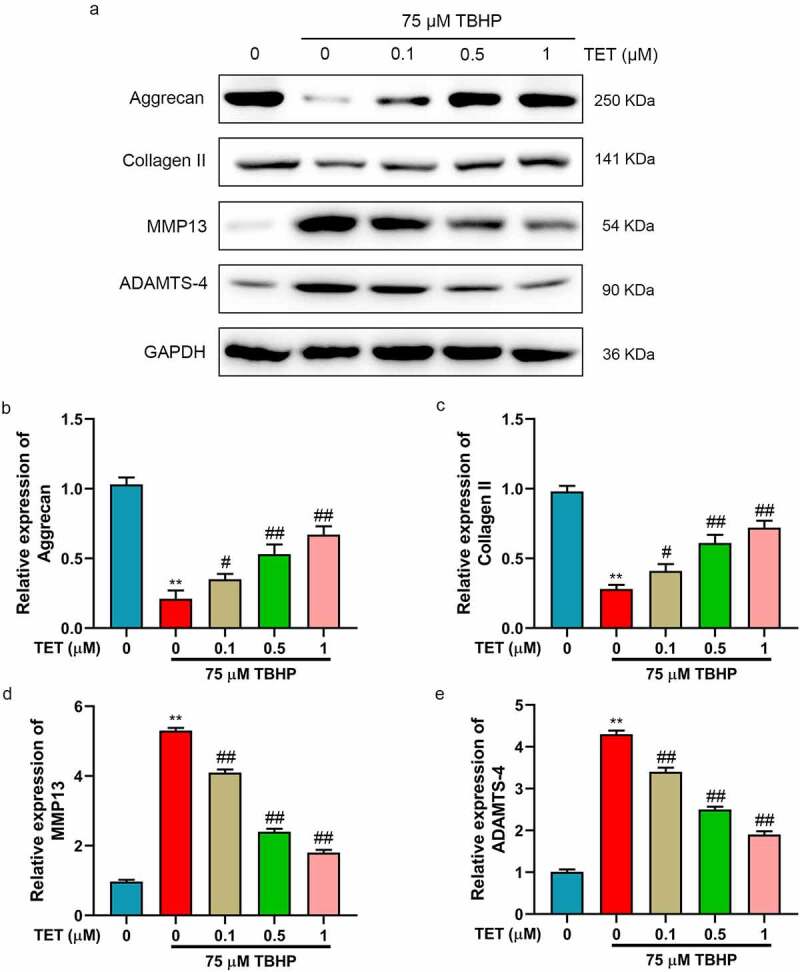
TET inhibited extracellular matrix (ECM) degradation in TBHP-treated NP cells. (a) Aggrecan, collagen II, MMP13, and ADAMTS4 expression were measured by Western blot. (b-e) The relative expression of aggrecan, collagen II, MMP13, and ADAMTS4 were showed in histograms. **p < 0.01 vs. control group, #*p* < 0.05 vs. TBHP group, ##*p* < 0.01 vs. TBHP group.

The loss of ECM is regarded as the main feature of IDD [6]. It was studied whether TET could increase ECM. Therefore, the expression of ECM-related markers (aggrecan, collagen II, MMP13, and ADAMTS-4) was detected by Western blot (Figure 3a). The results showed that aggrecan and collagen II expression were significantly increased in TBHP-treated NP cells, and which were inhibited by TET (Figure 3b,c). Moreover, TBHP treatment significantly increased the expression of MMP13 and ADAMTS-4, while TET reversed these effects (Figure 3d,e). These results suggested that TET could restored the ECM degradation caused by TBHP in NP cells.

### TET inhibited oxidative stress, apoptosis, and ECM degradation in TBHP-treated NP cells by promoting autophagy

**Figure 4.**
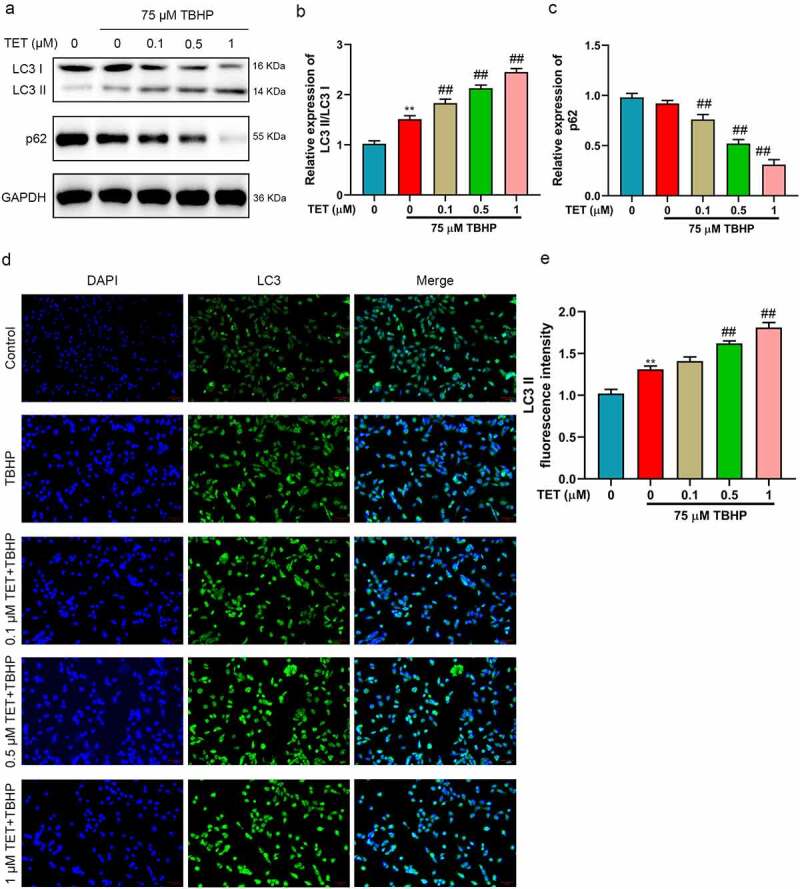
TET promoted autophagy in TBHP-treated NP cells. (a) LC3 and p62 expression were detected by Western blot. (b-c) The relative expression of LC3II/LC3I and p62 were showed in histograms. (d) LC3 expression was confirmed by immunofluorescence assay. (e) LC3II fluorescence intensity was showed in a histogram. **p < 0.01 vs. control group, ##*p* < 0.01 vs. TBHP group.

**Figure 5.**
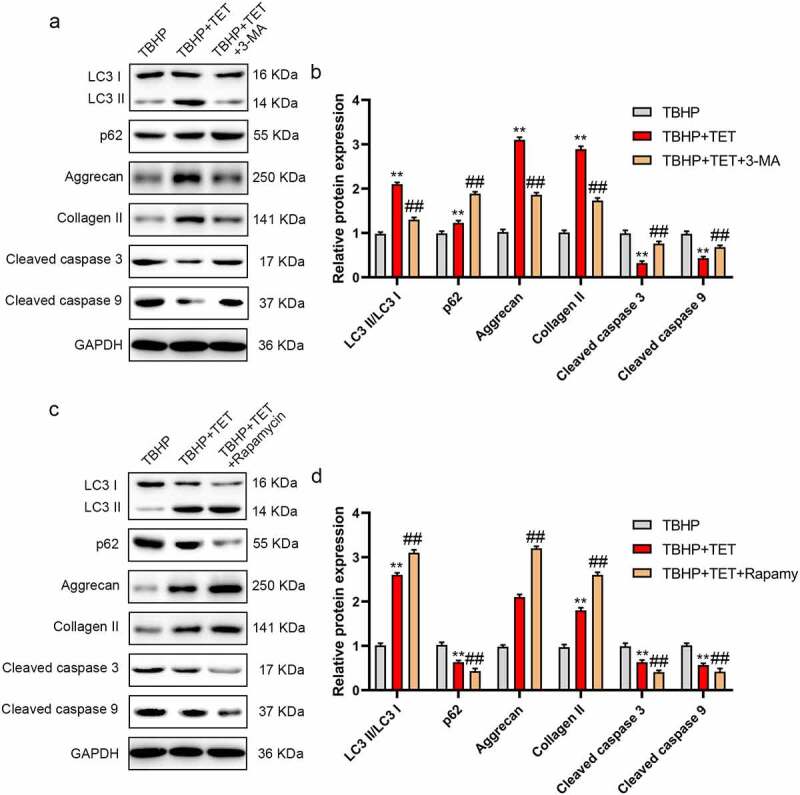
TET attenuated NP cells injury induced by TBHP through activating autophagy. (a) NP cells were treated with TBHP, TBHP+TET, or TBHP+TET+3-MA, and then LC3, p63, aggrecan, Collagen II, MMP13, and ADAMTS4 expression were measured by Western blot. (b) The relative expression of LC3II/LC3I, p62, aggrecan, collagen II, cleaved caspase 3 and cleaved caspase 9 were showed in a histogram. (c) NP cells were treated with TBHP, TBHP+TET, or TBHP+TET+Rapamycin, and then LC3, p63, aggrecan, Collagen II, MMP13, and ADAMTS4 expression were measured by Western blot. (d) The relative expression of LC3II/LC3I, p62, aggrecan, collagen II, cleaved caspase 3 and cleaved caspase 9 were showed in a histogram. **p < 0.01 vs. control group, ##*p* < 0.01 vs. TBHP group.

Previous studies have shown that TET could induce autophagy [25]. Moreover, studies have reported that the enhancement of autophagy could alleviate IDD [16]. We hypothesized that TET improved IDD through activating autophagy. In this study, we explored the effect of TET on autophagy in TBHP-induced NP cells. The results showed that TBHP increased the ratio of LC3 I/LC3 II, did not significantly change the expression of p62, and TET further enhanced TBHP activated autophagy (upregulating the ratio of LC3 I/LC3 II and inhibiting p62 expression) (Figure 4a,c). Similarly, the result of immunofluorescence for the LC3 expression were consistent with that of Western blot (Figure 4d,e). To further verify whether TET inhibited TBHP-induced oxidative stress, apoptosis and ECM degradation in NP cells by modulating autophagy, 3-MA (autophagy inhibitor) and rapamycin (autophagy agonist) were applied. 3-MA prevented TET-mediated autophagy and blocked the protective effect (increase of aggrecan and collagen II, decrease of cleaved caspase-3 and cleaved caspase-9) of TET on NP cells (Figure 5a,b). Rapamycin enhanced the protective effect of TET on NP cells and further increased autophagy (Figure 5c,d). These results confirmed that TET protected NP cells by inducing autophagy.

### *TET improved IDD in* vivo

**Figure 6.**
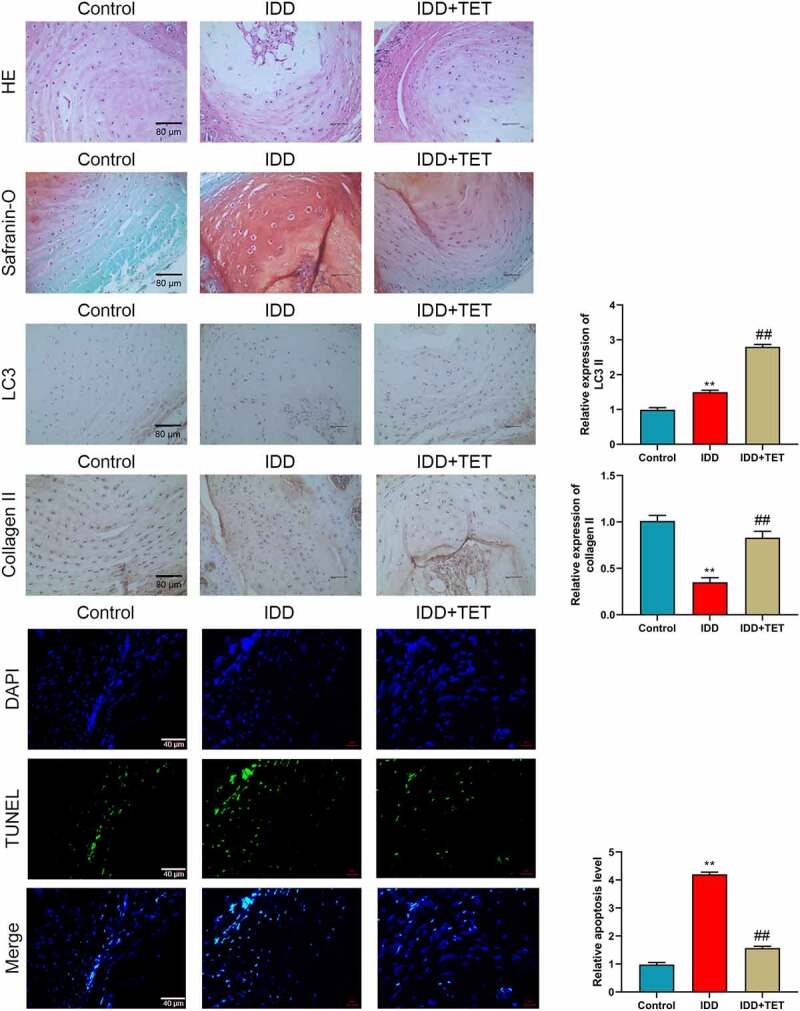
TET delayed rat intervertebral disc degeneration induced by puncture in annulus fibrosus. (a-b) The pathological changes of intervertebral disc were detected by HE and Safranin-O staining. (c-f) LC3 and Collagen II expression in intervertebral disc were measured by immunohistochemistry, and the relative expression were showed in histograms. (g) The apoptosis was assessed by TUNEL assay. (h) Relative apoptosis level was showed in a histogram. **p < 0.01 vs. control group, ##*p* < 0.01 vs. TBHP group.

The improvement effect of TET on IDD was studied in vivo. A rat IDD model was constructed by AF puncture. The results of HE and SO staining showed that puncture-induced rupture of annulus fibrosus, and NP cells and ECM were significantly reduced in IDD model group (Figure 6a,b). TET treatment significantly improved the pathological changes of IDD induced by puncture (Figure 6a,b). Similarly, puncture increased the number of LC3 II positive cells and decreased the number of collagen II positive cells compared with the control group (Figure 6c,f). In TET treatment group, the number of LC3 II positive cells were further increased and the number of collagen II positive cells was also significantly increased (Figure 6c,f). Moreover, the apoptosis of IVD cells was detected by TUNEL assay. The results indicated that less TUNEL apoptotic cells were observed in the TET treatment group than that in the IDD group (Figure 6g,h). These demonstrated that TET resisted NP cell apoptosis and ECM degradation and improved IDD by promoting autophagy.

## Discussion

IVD is a composite structure composed of central NP, peripheral AF and upper and lower endplate cartilage. NP is located in the center of IVD, which play an important role in maintaining the physiological morphology of IVD [26]. Rupture of AF, apoptosis of NP cells and reduction of ECM synthesis are all important factors to accelerate IDD [27]. In clinic, surgeons can use physical therapy to repair injury induced by IDD and relieve patients’ pain, but the treatment cannot prevent further degeneration of IVD and its complications [28,29]. Therefore, it is of great clinical significance to explore new drugs that can effectively slow down IDD. In this study, TET was found for the first time to alleviate TBHP-induced oxidative stress, apoptosis, and ECM digestion of NP cells by activating autophagy. Moreover, in *vivo*, the results further confirmed that TET delayed IDD induced by AF puncture through autophagy.

Oxidative stress induced by IDD could promote the apoptosis of nucleus pulposus cells, reduce the number of NP cells, and further accelerate the progression of IDD [24,30,31]. It has been found that inhibiting oxidative stress and apoptosis could delay IDD [24,32]. In normal NP cells, the cells secrete rich ECM components. However, in the progression of IDD, the synthesis and catabolism of ECM in NP cells are unbalanced, which is mainly manifested in the gradual decrease of the expression of collagen II and proteoglycan, resulting in the change of morphology and structure of IVD and accelerating degeneration [6,33]. MMPs and ADAMTSs are the degradation enzymes of collagen and proteoglycan, respectively [6]. MMP3, MMP13, ADAMTS4 and ADAMTS5 are up-regulated in IDD [34]. However, the inactivation or knockout of these enzymes could relieve the degradation of ECM and delay IDD [35–37]. Therefore, oxidative stress, NP cell apoptosis and ECM degradation were evaluated in this study.

Autophagy is a protective mechanism for eukaryotic cells to maintain intracellular homeostasis [38]. It degrades unnecessary and damaged organelles and proteins in cells through lysosomes, so as to respond to external stimuli and meet internal metabolic needs [38]. At present, most studies have shown that the activation of autophagy can delay IDD [39–41]. It is reported that curcumin could promote autophagy in NP cells through AMPK/mTOR/ULK1 signaling pathway, and then reduce ROS, mitochondrial disorder, NP cell apoptosis, and ECM degradation. IL-17A could accelerate IDD, which is closely related to its induced inhibition of autophagy [21]. Inhibition of autophagy can enhance apoptosis and ROS level of NP cells induced by cyclic mechanical tension (CMT), thus accelerating IDD [16]. Therefore, regulating the activation of autophagy might be an effective measure for the treatment of IDD.

TET, a dibenzyl quinoline alkaloid, is extracted from *Stephania tetrandra S. Moore*, which possesses a variety of pharmacological effects [17]. TET promoted autophagy of tumor cells and induced cell death, which was related to the inhibition of PKC-a and the inactivation of mTOR [42]. It has been found that TET is an autophagy agonist in a variety of cell lines (including tumor and non-tumor), which could even induce stronger autophagy than rapamycin [43]. However, whether TET could improve IDD by inducing autophagy is unknown. In our study, TET could effectively reduce oxidative stress, apoptosis, and ECM degradation in TBHP-treated NP cells, and promote autophagy. To verify whether TET attenuates TBHP’ damage to NP cells by inducing autophagy, rapamycin and 3-MA were used. Our results suggested that 3-MA reversed TET-induced autophagy through amplifying the ratio of LC3 I/LC3 II and reducing p62 expression in TBHP-treated NP cells. Correspondingly, after 3-MA treatment, the inhibition of TET on NP cell apoptosis and ECM degradation was blocked, which was confirmed by the downregulation of aggrecan and collagen II and the upregulation of cleaved caspase-3,9. On the contrary, rapamycin had the opposite effect on TET-treated NP cells. Compared with 3-MA, rapamycin increased TET-induced autophagy and further attenuated TBHP-induced NP cell injury. In addition, the effect of TET on IDD was explored in *vivo*. The rat IDD model was constructed by AF puncture, which was confirmed by HE and SO staining. Immunohistochemical results indicated that the number of LC3 and collagen II positive cells was reduced in IDD model group compared with the control group, and the results from TUNEL staining showed that NP cells apoptosis was enhanced in IDD model group. Besides, TET treatment significantly improved IDD by increasing LC3 and collagen II expression and inhibiting apoptosis.

In addition, TET could regulate autophagy through a variety of signaling pathways [25,44–46], and our study lacked the exploration of these signaling pathways. We will further study the role of TET in the treatment of IDD in the future.

## Conclusion

Our data demonstrated that TET inhibited oxidative stress, apoptosis, and ECM degradation in TBHP-treated NP cells by increasing autophagy. Moreover, in *vivo*, the results also verified that TET improved IDD via activating autophagy. Our study indicated that TET might be used in the treatment of IDD in the future.

## Data Availability

The datasets used and/or analyzed during the current study are available from the corresponding author on reasonable request.
